# Mechanical and Fatigue Properties of a Laser Powder Bed Fusion Ti6Al4V Alloy: A Review

**DOI:** 10.3390/ma19214752

**Published:** 2026-11-09

**Authors:** Žiga Žnidarič, Branko Nečemer, Srečko Glodež

**Affiliations:** Faculty of Mechanical Engineering, University of Maribor, 2000 Maribor, Slovenia; ziga.znidaric@um.si (Ž.Ž.); branko.necemer@um.si (B.N.)

**Keywords:** Ti6Al4V, Laser Powder Bed Fusion (LPBF), mechanical properties, fatigue

## Abstract

Laser Powder Bed Fusion (LPBF) enables the manufacturing of complex Ti6Al4V components, but the resulting mechanical and fatigue responses are condition-dependent. This critical narrative review examines how alloy grade and interstitial chemistry, powder history, processing, microstructure, residual stress, defect population, surface condition and post-processing affect quasi-static behaviour and low-, high- and very-high-cycle fatigue behaviour. Reported properties are compared with the associated material and test data, including surface condition, build orientation, stress ratio, loading mode, stress definition and run-out criterion. The review distinguishes finite-life fatigue strength from a demonstrated fatigue limit and separates stress-life data from strain-life and crack-growth parameters. The resulting condition-specific synthesis is intended to support engineering comparison and numerical modelling without treating LPBF Ti6Al4V as a single universal material dataset.

## 1. Introduction

Ti6Al4V is a widely used and studied titanium alloy for additive manufacturing because of its high specific strength, corrosion resistance, biocompatibility, and established aerospace and biomedical use. Laser Powder Bed Fusion (LPBF) is attractive for producing complex components, but their mechanical properties remain strongly dependent on processing and microstructural conditions [1].

The rapid thermal history of LPBF produces steep thermal gradients and residual stresses [2,3]. Insufficient melt-track overlap can cause irregular lack-of-fusion defects [4,5,6], whereas excessive local energy input can cause evaporation and keyhole porosity [5,7]. Surface irregularities occur from partially fused particles, melt-track geometry and the orientation of the manufactured surface [8,9]. Because of this, their morphology and location vary within a component.

Static tensile strength alone is insufficient for reliable component design. Studies have shown that LPBF Ti6Al4V can reach high yield and ultimate strengths [10,11], but additional mechanisms govern fatigue resistance. Rough surface valleys and adhered particles promote surface crack initiation [12], while near-surface defects can be particularly detrimental because of their interaction with the free surface [13].

Published fatigue datasets differ in alloy chemistry, build orientation, specimen geometry, surface and post-processing condition, loading mode, stress ratio, test frequency and the adopted fatigue-life or run-out criterion [14,15,16]. Consequently, reported fatigue strengths should not be directly compared unless the material condition, stress definition, loading conditions, surface state and life criterion are comparable.

The objective of this review is to connect LPBF processing, microstructure, defects, build orientation, and post-processing with the static and cyclic response of Ti6Al4V alloy. Emphasis is placed on the fatigue life, life scatter, and the conditions required for defensible comparison of published data.

Recent reviews already provide broad process–microstructure–fatigue syntheses for LPBF Ti6Al4V and critical assessments of defect-controlled HCF (high-cycle fatigue) and VHCF (very-high-cycle fatigue) [17,18]. The distinctive purpose of the present review is therefore not to repeat that broad coverage, but to organise condition-specific quantitative data for comparison and modelling. It distinguishes Grade 5 and Grade 23 chemistry, separates maximum stress, stress amplitude, and stress range, records stress ratio, loading mode, and run-out definitions where reported, and collects tensile, cyclic stress–strain, strain-life, and fatigue-crack-growth parameters.

Li and Affolter [17] focus on internal defects and HCF/VHCF in LPBF Ti6Al4V, whereas Idury et al. [19] compare defect–fatigue relationships and post-processing across several LPBF alloy families. Vafaei et al. [18] provide a broad Ti6Al4V process–microstructure–fatigue synthesis that also covers fatigue modelling, and Cheng et al. [20] review process-microstructure-defect-property relationships across aerospace titanium alloys. The present review complements these scopes through condition-specific quantitative comparisons and by assessing when published static, stress-life, strain-life, and crack-growth parameters can be transferred to engineering analyses, and when differences in chemistry, surface condition, defects, orientation, or post-processing prevent such transfer.

## 2. Review Scope and Methodology

This article is a critical narrative review rather than a systematic review. The PRISMA framework was therefore not formally applied; however, a structured literature-search and selection procedure was used to improve the transparency and reproducibility of the review. It focuses on peer-reviewed studies of LPBF Ti6Al4V that report processing–structure–property relationships, quasi-static tensile behaviour, stress-life or strain-life data and the effects of post-processing. Electron-beam powder-bed-fusion studies or other related manufacturing techniques are included only when they provide a clearly identified mechanism that is relevant and transferable to LPBF, and their results are not pooled with LPBF data.

The literature search was conducted using Web of Science, Scopus, and UM:NIK, the University of Maribor search tool. The final search was completed in October 2026. Searches used combinations of the terms “Ti6Al4V”, “laser powder bed fusion” or “selective laser melting”, “fatigue”, “HCF”, “VHCF”, “defect”, “surface condition”, “HIP (hot isostatic pressing)”, “powder reuse”, “oxygen”, and “nitrogen”. Eligible publications were peer-reviewed journal articles and reviews that reported directly relevant LPBF Ti6Al4V evidence, including material chemistry or grade, process and post-process condition, microstructure, defects, quasi-static properties, fatigue behaviour, crack initiation, or crack growth. Studies of other additive manufacturing processes were retained only when they provided clearly labelled mechanistic evidence. Conference papers, theses, non-peer-reviewed web sources, and studies lacking sufficient relevance to the defined scope were excluded from the review. No study was excluded solely because its results differed from the general trend, and no lower date limit was set for the publication date. Additional publications incorporated during revisions were identified through targeted follow-up searches and reference list screening.

The widely used abbreviation LPBF is retained throughout this review. Historical terms such as selective laser melting (SLM) and direct metal laser sintering (DMLS) are used only when reproducing terminology from the cited literature. Grade 5 and Grade 23 extra-low-interstitial (ELI) conditions are distinguished wherever material chemistry is reported [21,22].

Quantitative comparisons prioritised primary experimental studies that reported the material grade or chemistry, manufacturing and post-processing conditions, surface state, build orientation, loading mode, stress ratio, stress definition, and fatigue life or run-out criterion. Particular emphasis was placed on studies that provided controlled comparisons of processing conditions, defect populations, surface treatments, or build orientations, along with experimental fatigue results. Where experimental conditions were insufficiently documented or incompatible, the results were retained for qualitative interpretation but excluded from direct quantitative comparison.

The review focuses primarily on room-temperature fatigue behaviour of dense LPBF Ti6Al4V specimens. It is not intended as an exhaustive fatigue database, and recently published large-scale datasets are not systematically analysed. Long-term thermal exposure and ageing effects on fatigue, application-specific biomedical and aerospace loading spectra, and fatigue validation at the component scale are also outside its scope.

The review first examines chemistry, processing, post-processing, and manufacturing defects. It then evaluates static properties, stress-life and strain-life behaviour, and minimum reporting requirements for transferable fatigue data.

## 3. Material, Processing, and Resulting Conditions

Ti6Al4V Grade 5 and ELI Grade 23 share the nominal 6Al4V designation but have different allowable composition and interstitial limits as presented in Table 1. For powder-bed-fused material, the maximum specified oxygen contents are 0.2 wt.% for Grade 5 and 0.13 wt.% for Grade 23, according to ASTM F2924 [22] and ASTM F3001 [21], respectively. Interstitial oxygen generally increases hardness and tensile strength while reducing ductility [23,24]. Dietrich et al. [24] reported an increase of approximately 30 MPa in ultimate tensile strength with increased atmospheric oxygen during LPBF, with nitrogen also varying, whereas the lower-oxygen condition exhibited better fatigue resistance. Quintana and Tong [14] reported higher tensile strengths for Grade 5 than Grade 23 specimens in both untreated and HIP-treated conditions. After HIP, smooth Grade 5 specimens also exhibited higher rotating-bending strength at 10^7^ cycles, whereas the notched fatigue strengths at 10^7^ cycles of the two grades were similar. Material grade alone is therefore insufficient to describe the expected mechanical performance. The measured oxygen content of the manufactured material should also be reported. Under the conditions investigated by Quintana and Tong [14], no measurable oxygen pickup occurred during printing or HIP.

Nitrogen is another interstitial element that must be distinguished from oxygen when alloy grade and powder history are reported. Both Grade 5 and Grade 23 permit a maximum nitrogen content of 0.05 wt.%. Grade 23 has tighter limits for oxygen, iron and hydrogen [21,22]. High-temperature X-ray diffraction experiments on stress-free Ti6Al4V powder showed that nitrogen and oxygen absorption produces residual lattice expansion. This study was not an LPBF or fatigue investigation and is used only as lattice-level evidence [25]. In conventionally processed Ti6Al4V, dissolved nitrogen increased strength but reduced ductility and fatigue resistance as its content increased [26]. These results should not be converted directly into LPBF design values, but they support reporting measured nitrogen together with oxygen when powder condition is investigated.

Powder reuse does not yield a universal property trend because the outcome depends on the reuse protocol, sieving and top-up procedures, atmospheric control, and the fraction of spatter returned to the feedstock. In an LPBF Grade 5 study, Soltani Tehrani et al. [27] linked repeated reuse to changes in oxygen content, flowability, and packing, as well as in the size of process defects. The resulting fatigue response was condition-dependent rather than monotonically degraded. Zhuo et al. [28] likewise reported that reused LPBF Ti6Al4V powder developed changes in particle morphology and oxygen uptake, with downstream changes in ductility associated with incomplete melting and defects. Under appropriate powder-reuse protocols, changes in mechanical properties are limited, and the corresponding effect on fatigue life is relatively small. A direct link between powder reuse and reduced fatigue performance should therefore be established only when it can be demonstrated experimentally under the specific reuse conditions.

### 3.1. LPBF Processing and Build Orientation

The LPBF process repeatedly melts and solidifies powder layers. Laser power, scan speed, hatch spacing and layer thickness determine melt-track overlap and melt-pool stability [4,5,6,7]. Scan strategy and powder-bed temperature also modify the thermal history, residual stress, and distortion [2,3]. Surface quality and microstructure therefore cannot be inferred from a single nominal energy-density value. As a first approximation, volumetric energy density is often described with:

(1) $$\eta =\frac{P}{v\cdot h\cdot t}$$

where *P* is laser power, *v* is scanning speed, *h* is hatch spacing, and *t* is layer thickness. The resulting energy density does not uniquely define the process conditions. Different parameter combinations at the same nominal volumetric energy density can produce different melt pools, cooling rates, residual stresses, and elongations [2,5]. Individual parameters should be reported to support reproducibility and comparison between studies.

If the energy is too low or the overlap is insufficient, lack-of-fusion defects can occur. In contrast, excessive energy can cause evaporation, keyhole instability, and rounded or elongated keyhole pores [4,5,7]. All these defects affect fatigue life differently, so relative density alone is an incomplete quality metric. Defect size, morphology, location and orientation also influence fatigue resistance [13].

Rapid LPBF cooling commonly transforms high-temperature β into acicular $\alpha^{\prime }$ martensite within elongated prior-β grains [29,30]. This microstructure and high dislocation density contribute to the high strength and comparatively limited ductility of the as-built alloy [30].

**Figure 1:**
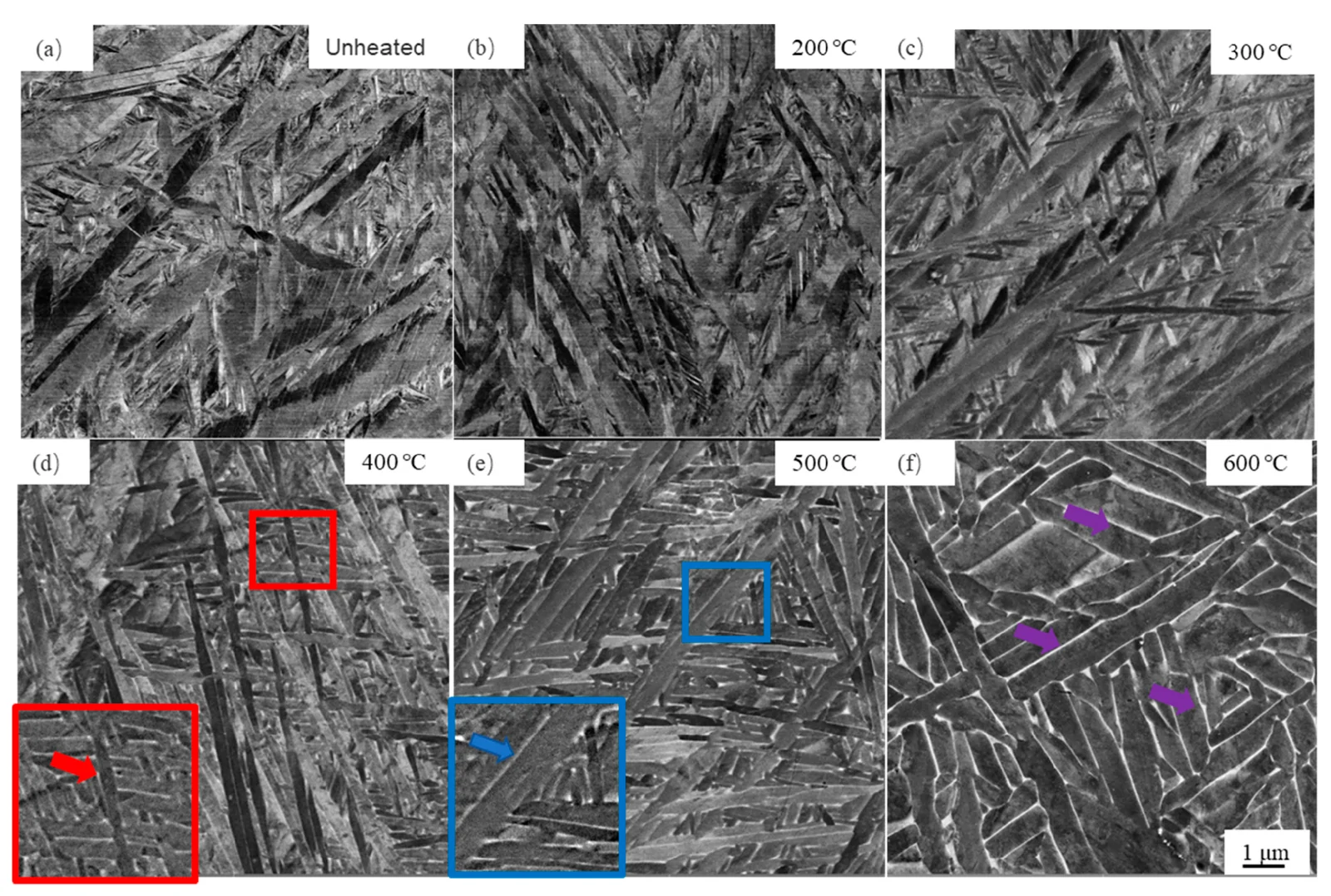
Effect of powder-bed preheating temperature on the microstructure of LPBF Ti6Al4V: (**a**) unheated; (**b**) 200 °C; (**c**) 300 °C; (**d**) 400 °C; (**e**) 500 °C; (**f**) 600 °C. Reproduced from Xing et al. [31] under the Creative Commons Attribution 4.0 licence.

Repeated reheating and partial remelting alter the dimensions and orientation of the acicular martensite [6,29]. Powder-bed preheating modifies and coarsens the microstructure, as illustrated in Figure 1. Depending on temperature and exposure time, preheating may promote partial decomposition of martensitic $\alpha^{\prime }$ [31]. The micrographs shown should not be interpreted as proof of complete transformation to an equilibrium α + β microstructure at every temperature.

The scan strategy can also affect the microstructure and, consequently, the mechanical response. It affects the heat-flow paths, residual stress, distortion, surface topography, and sometimes porosity. A 90° alternating strategy reduced the residual-stress accumulation in the conditions tested by Ali et al. [3], while Valente et al. observed lower surface roughness with a line strategy than with an island strategy [8]. These effects are process-specific rather than universal.

**Figure 2:**
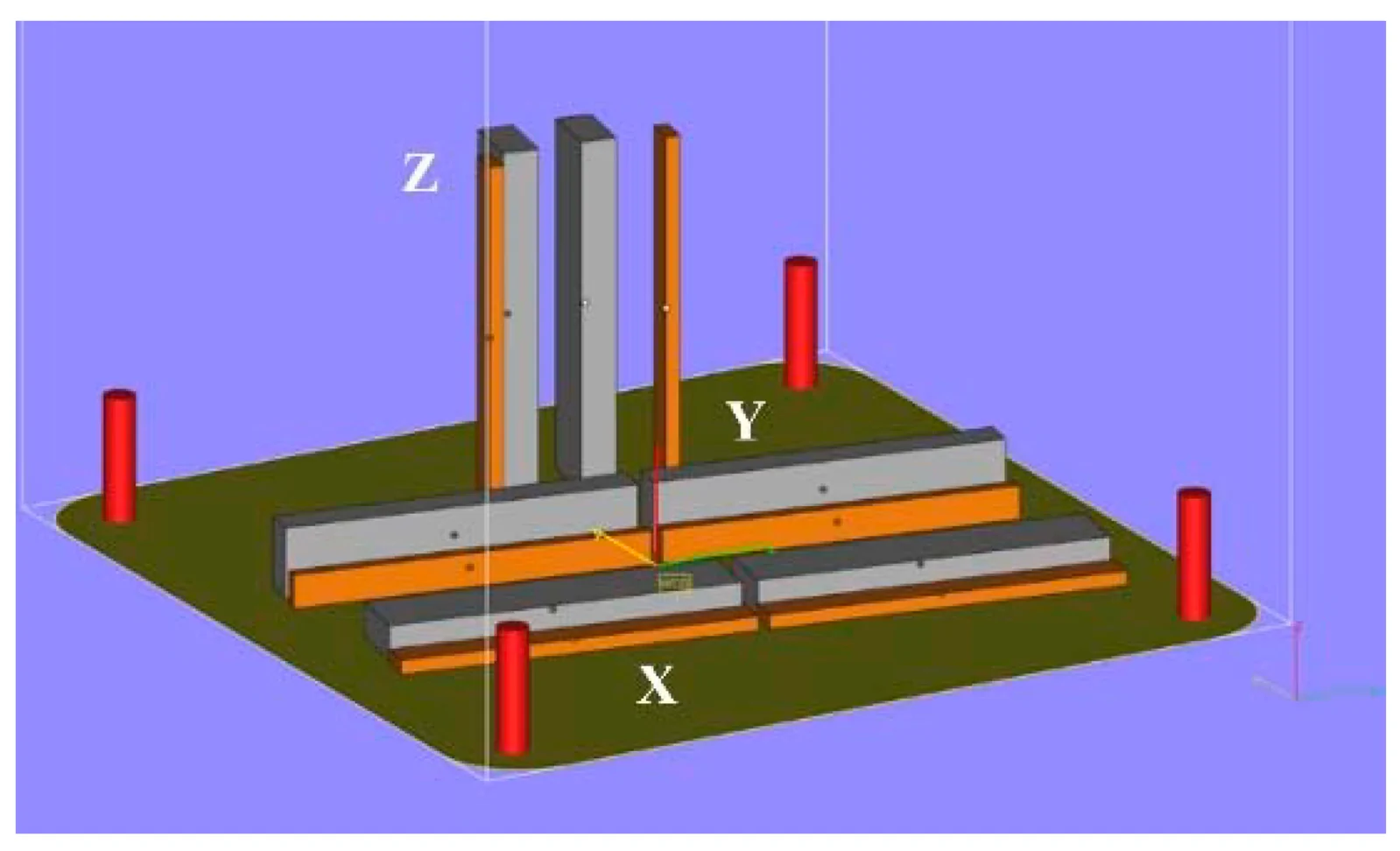
Build orientations and specimen arrangement used to investigate anisotropy in LPBF Ti6Al4V. Reproduced from Hartunian and Eshraghi [32] under the Creative Commons Attribution 4.0 licence.

Build orientation changes the alignment of layers, prior-β grains, surfaces, and defects relative to the applied load. A pronounced orientation effect on ductility and fracture mode was observed by Simonelli et al. [10], while Hartunian and Eshraghi [32] reported lower tensile strength for vertically built specimens than in the investigated horizontal direction. The relationship between the specimen axis and the build direction is illustrated schematically in Figure 2.

Build orientation also changes the local thermal history and phase constitution. Xie et al. [33] related orientation-dependent tensile strength and ductility to interlayer remelting and the relative amount of α and $\alpha^{\prime }$. Agius et al. [34] observed anisotropy and cyclic softening during strain-controlled loading, with residual stresses contributing to the cyclic response. Build orientation affects tensile strength and stress-life fatigue performance, whereas Sun et al. [35] observed a comparatively small effect on crack-growth behaviour. Sun’s axial S–N tests compared 0° and 90°; the 45° specimens were included in tensile and crack-growth tests. Yang et al. [9] additionally demonstrated orientation-dependent differences in surface and near-surface quality for specimens built between 0° and 90°. Orientation effects should therefore be interpreted as condition-specific rather than universally applicable [9,35].

### 3.2. Residual Stress in LPBF Ti6Al4V

Residual stress develops because the highly localised LPBF heat source produces steep thermal gradients, while the surrounding solid material and the build plate constrain contraction of each cooling track and layer. Repeated heating, melting, solidification and cooling redistribute this stress as the part height increases. The final field is therefore affected by scan strategy, process parameters, component geometry, support constraint, and build orientation rather than by energy density alone. Levkulich et al. [36] measured tensile stresses at deposit surfaces and compressive stresses in the bulk material, and also showed that laser power, scan speed, build height, plan area, and substrate condition altered the stress and distortion response. Recent work on thin-walled LPBF Ti6Al4V further confirms that stress-relief annealing alters residual stress alongside the evolving microstructure [37].

The measurement method determines which part of the field is observed. Laboratory X-ray diffraction is non-destructive and well-suited to near-surface stresses but has limited penetration. High-energy synchrotron X-ray diffraction and neutron diffraction can probe the interior and resolve spatial variations, although they require specialised facilities and careful treatment of texture, phase-specific lattice parameters and the stress-free reference. Gloaguen et al. [38] used neutron diffraction to map the full stress tensor at different depths and positions in LPBF Ti6Al4V cubes. Incremental hole drilling provides a local depth-dependent estimate but is semi-destructive and averages the released strain over the sampled region. The contour method is destructive and maps one stress component over a cut plane. Levkulich et al. [36] combined X-ray diffraction, hole drilling and the contour method, illustrating why surface and bulk measurements should not be treated as interchangeable.

Mechanically, a near-surface tensile residual stress increases the local mean stress at a potential crack-initiation site. Assuming elastic superposition and an approximately stable residual-stress field, the local stress ratio can be expressed as [39]:

(2) $$R_{local}=\frac{\left(\sigma_{min}+\sigma_{res}\right)}{\left(\sigma_{max}+\sigma_{res}\right)}$$

where $\sigma_{min}$ and $\sigma_{max}$ are the minimum and maximum applied stresses and $\sigma_{res}$ is the residual-stress component acting normal to the prospective crack plane. Tensile residual stress can increase crack opening and reduce crack closure without changing the applied stress range [40]. Conversely, compressive residual stress can reduce the local tensile driving force, providing that the compressive layer extends sufficiently deep and remains stable during cyclic loading. Local yielding, high applied stress amplitudes or crack growth through the residual-stress gradient may reduce this benefit as stresses redistribute or relax. The local stress ratio is an elastic estimate and should not be confused with a crack-closure-corrected effective stress-intensity factor ratio [40]. Mishurova et al. [41] separated the contributions of residual stress, defects and microstructure in LPBF Ti6Al4V tested at 300 °C. They reported reduced fatigue performance associated with tensile surface residual stresses in the as-built condition, particularly at low strain amplitudes. These results demonstrate the importance of residual stress but do not establish its stability during room temperature fatigue cycling.

Thermal stress relief reduces residual stress but simultaneously tempers or decomposes martensitic $\alpha^{\prime }$ depending on temperature and time. HIP also relaxes residual stresses, changes the microstructure, and closes suitable enclosed pores. Therefore, its fatigue benefit cannot be attributed solely to stress relaxation. Zou et al. [42] measured stress relief after fewer than 20 progressively increased slow elastic loading cycles in PBF-LB Ti6Al4V. This mechanical stress-relief procedure is not equivalent to conventional fatigue testing. Within the literature examined in this review, no directly comparable study was identified that systematically measured the evolution of surface or bulk residual stress in LPBF Ti6Al4V at defined intervals during conventional fatigue cycling. This represents a limitation of the available evidence rather than proof that such measurements do not exist. Residual-stress relaxation should therefore be evaluated for the relevant loading history rather than assumed from the initial stress state.

Taken together, residual stress must be interpreted in light of the build orientation, geometry, defect location, surface condition, and post-processing route. A near-surface tensile measurement may be directly relevant to an as-built surface-controlled specimen. In contrast, the same value may be less decisive after machining, HIP or a shift to internal initiation. Reporting the measurement method, direction, depth, and component condition is consequently essential for comparing residual-stress data and for determining whether a fatigue difference is caused by stress, microstructure, defects, or their interaction.

## 4. Post-Processing of LPBF Ti6Al4V

Post-processing routes modify different parts of the LPBF material condition. Stress relief and annealing reduce residual stress and change the $\alpha^{\prime }$ to α + β microstructure [43,44]. HIP additionally closes enclosed internal pores but does not remove the rough external surface [45]. Machining, polishing, and chemical or electrochemical finishing primarily modify the external surface [15,46]. Combined routes may be required when both enclosed porosity and surface initiation are critical [47].

### 4.1. Stress Relief and Annealing

Stress relief and annealing reduce residual stresses and progressively decompose the as-built $\alpha^{\prime }$ martensite into α + β. Increasing the process temperature and holding time generally lowers hardness and strength while increasing ductility, but the magnitude also depends on the cooling rate and microstructural coarsening [43]. Carefully selected high-temperature treatments can produce bimodal microstructures with improved tensile ductility, although the resulting properties depend on the treatment conditions [48].

### 4.2. Hot Isostatic Pressing

Hot isostatic pressing (HIP) combines elevated temperature and isostatic pressure. It can close enclosed internal pores [49], while simultaneously transforming the martensitic microstructure and reducing residual stress. Qiu et al. [44] reported lower strength but substantially improved ductility after HIP. HIP does not reliably close surface-connected defects and does not remove the rough as-built surface, so surface finishing may still be required when initiation remains surface-controlled [45,50]. Lower-temperature/high-pressure HIP can preserve a fine microstructure [51], although effective pore closure depends on the combined effects of temperature, pressure, holding time, and defect characteristics [52]. Gas-filled pores may regrow during later thermal exposure, as demonstrated mechanistically for electron-beam powder-bed-fused Ti6Al4V [53].

### 4.3. Surface Post-Processing

Parts manufactured with LPBF have a rough surface with adhered particles, melt-track irregularities, valleys, and surface-connected pores that often control fatigue crack initiation. Machining and polishing are therefore commonplace to produce a smoother finish on accessible surfaces. Peening and laser shock peening can also introduce compressive residual stresses on the part surface, but their benefits depend on coverage and whether deep valleys remain [54]. Cutolo et al. [15] found that retained down-facing valleys limited the fatigue benefit of electroplasma polishing despite improved overall roughness. The main drawback of these processes is that they require access to the surface being modified. Chemical etching and electropolishing may be suitable when access for conventional machining is limited, depending on the geometry. Jamshidi et al. [46] showed that HIP and surface treatments address different defect types. Material removal must be controlled because excessive etching can change dimensions or expose subsurface pores [47].

Recent LPBF Ti6Al4V studies reinforce the need to separate surface geometry from bulk material condition. Melle et al. [55] showed that chemical etching progressively improved fatigue performance, but that large surface-connected cavities could remain life-controlling. Rogers et al. [56] quantified a strong dependence of fatigue life on the orientation of surface roughness, with the removal of the downward-facing rough surface increasing life under their tested conditions. These results support reporting the state of the post-processed surface and the depth of material removal, rather than describing a specimen only as etched, polished, or machined.

### 4.4. Combined Post-Processing Routes

**Figure 3:**
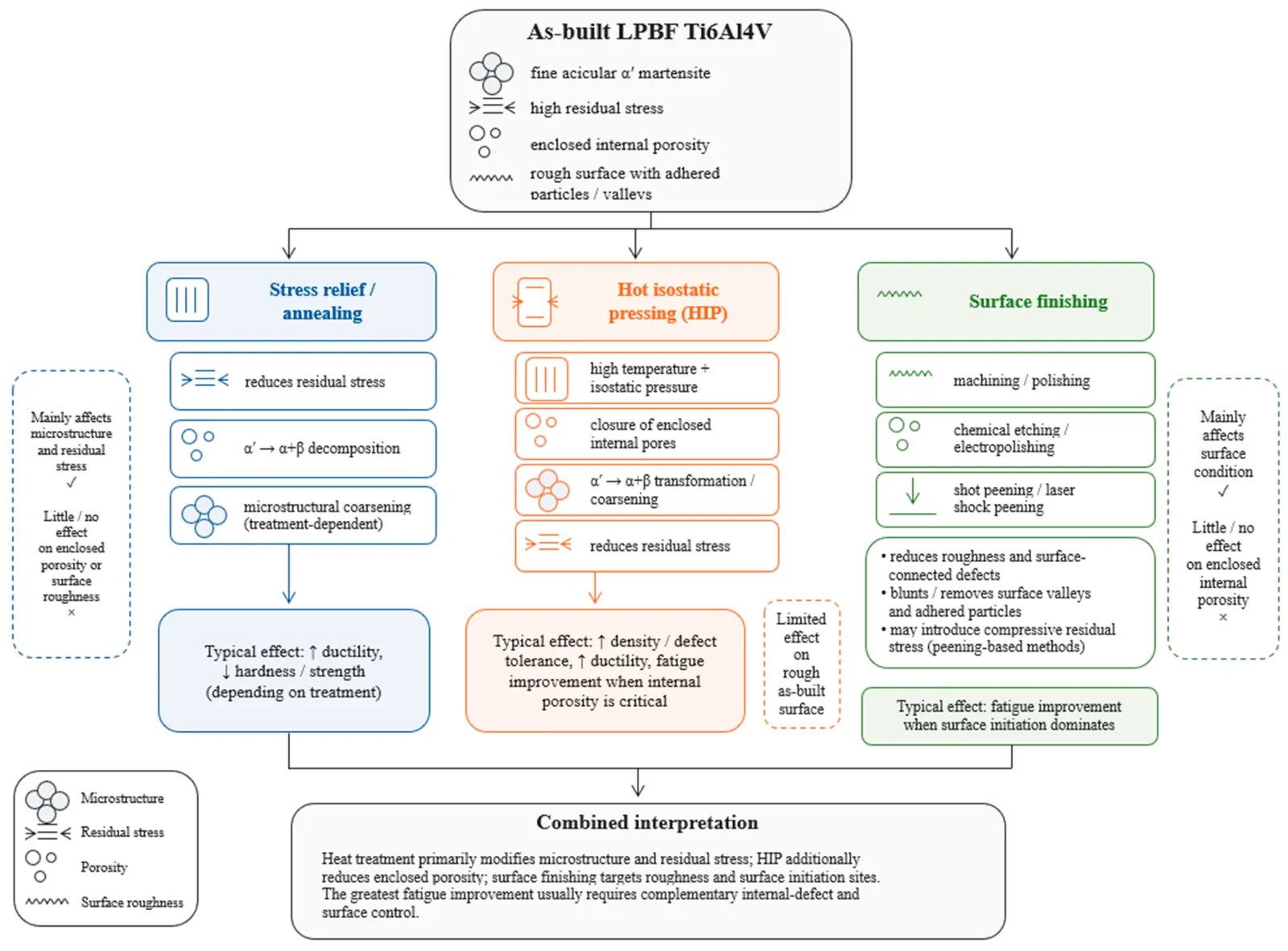
Principal mechanisms by which post-processing modifies the material condition of LPBF Ti6Al4V specimens. Arrows link the treatment routes to their principal effects and combined interpretation.

Combining internal-defect control with surface finishing can improve fatigue performance. Hills et al. [47] showed that HIP combined with controlled chemical etching improved the material’s fatigue strength by reducing internal porosity and surface irregularities. The optimum route, however, remains application-specific. Machining is effective when the surfaces are accessible, whereas chemical and electrochemical treatments may be needed for inaccessible surfaces. The distinct effects of the post-processing routes are compared in Table 2 and summarised in Figure 3.

## 5. Manufacturing Defects

Manufacturing defects reduce fatigue strength and increase life scatter, but their effects cannot be represented by relative density alone. Processing studies identify distinct populations of lack-of-fusion, keyhole, and gas-related defects [4,5,57,58]. Fatigue severity depends on defect size, morphology and location relative to the free surface [13,50,59]. The following discussion therefore separates defect formation from defect severity. More broadly, defect–fatigue assessments show that defect size, morphology and location must be considered together rather than replaced by a single porosity fraction [60].

### 5.1. Defect Formation and Morphology

Insufficient local energy input or melt-track overlap produces irregular lack-of-fusion defects that may contain unmelted powder and align with layer or track boundaries [4,57]. Their sharp morphology and large projected area can make them more damaging than smoother pores of similar volume, and Gong et al. reported substantial tensile and fatigue losses even at relatively low lack-of-fusion content [50,57]. Excessive local energy input can instead promote keyhole instability and pore formation [5,57]. The severity of a keyhole pore still depends on its size, elongation, clustering, proximity to the surface and surrounding microstructure [61].

Rounded pores may arise from trapped gas or keyhole-related melt-pool instability. Morphology alone does not uniquely identify their origin. Their fatigue severity depends on size, shape and location. Sharp lack of fusion defects can be especially damaging. Nevertheless, a large near-surface gas pore or a cluster of them can still affect high-cycle fatigue. This is why classifying the defect population by formation mechanism and morphology is more informative than reporting a single porosity percentage [4,5,58,59].

### 5.2. Defect Severity: Size, Orientation, and Location

For fatigue assessment, four defect classes should be distinguished. Surface roughness denotes distributed geometric irregularities of the external surface. Surface-breaking defects are pores, lack-of-fusion defects or cracks that intersect the free surface. Near-surface defects are subsurface discontinuities whose stress field interacts with the free surface, and internal defects are sufficiently embedded to behave as internal discontinuities. Surface, near-surface, and internal defects should not be treated as mechanically equivalent because their locations relative to the free surface affect fatigue severity [13,59].

Once a defect is present, its fatigue severity depends on its projected size, morphology, orientation relative to the maximum principal stress, and position relative to the free surface [13,57,59]. Equal total porosity or relative density, therefore, does not imply equal fatigue resistance, because a large irregular defect can be more damaging than numerous small spherical pores. A practical measure is the square root of the defect area:

(3) $$\sqrt{area}$$

The parameter provides a practical link between defect geometry and fatigue assessment by representing the square root of the defect area projected perpendicular to the loading direction. Its value is therefore dependent on the loading direction and should be interpreted together with the defect location. Masuo et al. [50] showed that internal, near-surface, and surface-connected defects of comparable projected size can exhibit different fatigue severities. Moreover, it is a scalar measure and cannot fully capture the three-dimensional morphology of irregular defects, particularly lack-of-fusion defects, nor the interaction between neighbouring discontinuities [13,59]. For quantitative fatigue assessment, the $\sqrt{area}$ value should therefore be reported together with its projection convention, defect depth and surface connectivity. Where available, XCT-based measurements of three-dimensional shape, orientation and neighbouring defects should also be included, particularly for irregular lack-of-fusion defects [13,59].

**Figure 4:**
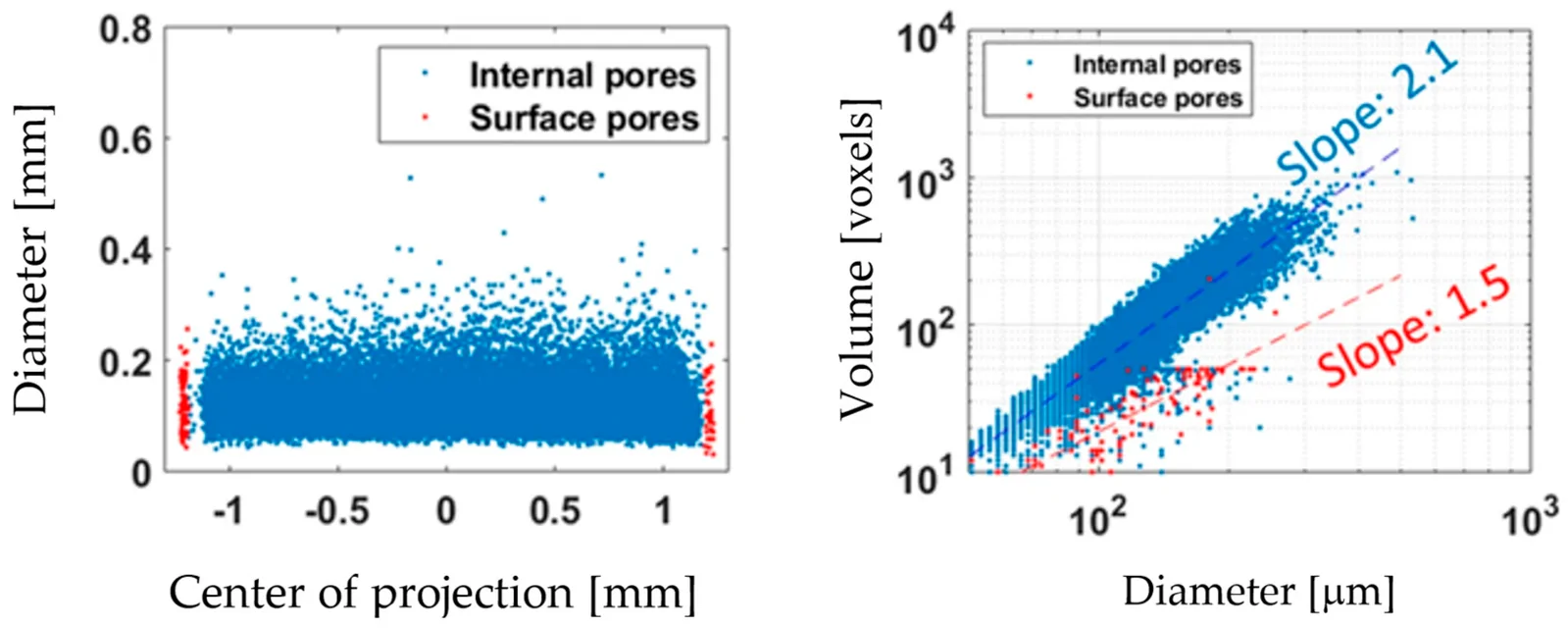
Characteristics and spatial distribution of internal and surface-connected pores in as-built LPBF Ti6Al4V. Reproduced from Moon et al. [59] under the Creative Commons Attribution 4.0 licence.

Near-surface defects can be especially damaging because of their interaction with the free surface (Figure 4). Tomography and crack-growth modelling by Hu et al. [13] showed particularly high severity of defects near the surface. Critical sites can be removed by surface finishing, but these procedures may also expose pores that were initially internal [62]. Fatigue studies should therefore report defect depth, surface connectivity and the defect population remaining after finishing, rather than treating size as the only descriptor. Figure 4 demonstrates that internal and surface-connected pores differ in their location and geometry.

Hu et al. [13] reported average $\sqrt{area}$ values of approximately 40 μm for lack-of-fusion defects and 20 μm for gas pores in the investigated LPBF Ti6Al4V specimens. The larger projected size and irregular morphology of lack-of-fusion defects contributed to their greater fatigue severity. Bonneric et al. [63] further showed that defect morphology influenced fatigue resistance when cracks initiated at the surface, whereas its influence was limited for internal defects under fully reversed loading (*R* = −1). These results demonstrate that defect size alone does not provide a complete description of fatigue severity.

Defect-based fatigue assessment can be performed using Kitagawa–Takahashi diagrams, with El-Haddad-type corrections describing the transition between small- and long-crack behaviour [64]. Murakami’s $\sqrt{area}$ approach instead relates fatigue resistance to projected defect size and material properties. Hu et al. [65] applied a Murakami-based assessment and extended the Kitagawa–Takahashi diagram to finite-life behaviour for SLM Ti6Al4V. However, these approaches require validation for the investigated material and defect population, particularly for irregular or interacting lack-of-fusion defects.

## 6. Quasi-Static Mechanical Behaviour of LPBF Ti6Al4V

The quasi-static response of LPBF Ti6Al4V reflects the combined effects of microstructure and defects introduced during manufacturing. Young’s modulus, yield strength, ultimate tensile strength and elongation must therefore be interpreted as a condition-specific set rather than as independent constants. Relevant conditions are oxygen content, printing parameters, build orientation, defect and surface state, type of post-processing used, specimen geometry and testing method [1,11].

### 6.1. Elastic Response

The Young’s modulus reported for dense LPBF Ti6Al4V generally varies less than its ultimate tensile strength and elongation. For the mechanically polished specimens in Table 3, Simonelli et al. [10] reported Young’s moduli of 112–119 GPa across build orientations and stress-relief conditions. Differences in material condition, porosity, crystallographic texture, specimen orientation, and measurement method should nevertheless be considered when comparing reported modulus values. Among the experimental studies included in this review, Poisson’s ratio was reported less frequently than Young’s modulus. When a conventional value is adopted for numerical modelling, it should be identified as an assumed input rather than a measured property of the specific LPBF material condition.

### 6.2. Tensile Strength and Ductility

LPBF Ti6Al4V usually exhibits a high yield and ultimate tensile strength. In many thermally untreated conditions, ultimate tensile strengths above 1100 MPa and elongations below 10% have been reported. The exact values, of course, depend strongly on the material’s chemistry, processing conditions, orientation, and defect content [1,11,44,66]. Rafi et al. [66] compared Ti6Al4V ELI manufactured by selective laser melting and electron beam melting. The SLM material consisted mainly of martensitic α’, while the electron-beam melted material consisted mostly of α + β. The different thermal histories and microstructures resulted in a difference in tensile response. The LPBF data from this study provide a useful benchmark, but the SLM and EBM datasets should remain separate, as their manufacturing processes and resulting microstructures differ. Mower and Long [11] compared the room-temperature tensile behaviour of LPBF Ti6Al4V with conventionally processed material. Their results showed that the LPBF material can achieve high yield and ultimate tensile strengths, while tensile ductility is more variable and sensitive to material quality. This distinction is important when comparing studies. Strength alone may indicate that the material meets a specification, while low or highly scattered elongation can reveal defects or a locally brittle material condition. Yield and ultimate strengths represent the resistance of the load-bearing material to plastic deformation, whereas elongation is more sensitive to premature localisation and fracture. A specimen can therefore retain a relatively high strength despite containing defects that substantially reduce its elongation. Qiu et al. [44] observed high strength and generally low elongation in the as-built condition. Following HIP, strength decreased while ductility improved considerably. This demonstrates that the material condition associated with the highest tensile strength is not necessarily optimal for applications requiring high ductility or damage tolerance.

### 6.3. Plastic Deformation and Strain Hardening

For non-linear numerical simulations, yield strength and ultimate tensile strength alone are usually insufficient. The stress–strain curve is required to accurately describe plastic deformation and local stress distribution. Useful literature datasets should therefore include:

- Young’s modulus
- Engineering stress–strain curve
- True stress–strain curve
- Elongation at fracture, with gauge length and strain definition
- Strain-hardening parameters.

The as-built martensitic material generally exhibits high initial strength but comparatively limited uniform plastic deformation. Kumar et al. [67] concluded that the mechanical response is governed by both the α′ microstructure and the larger-scale columnar prior-β-grain arrangement. Moridi et al. [68] investigated deformation using in situ tensile testing, electron microscopy, electron backscatter diffraction and digital image correlation. Strain localisation occurred within and along the boundaries of the martensitic structure. Microscopic void formations and coalescence were observed even though the overall tensile response appeared comparatively brittle. This demonstrated that the macroscopic fracture appearance should not automatically be interpreted as evidence of a completely cleavage-controlled fracture mechanism. Heat-treated material usually exhibits a lower yield strength but greater strain-hardening capability and ductility. Using in situ high-energy X-ray diffraction, Zhang et al. [69] found that decomposition of martensitic α’ into α + β reduced the material’s yield strength. The investigated 730 °C and 900 °C/2 h heat-treated conditions exhibited greater work hardening and elongation than the as-built condition. For finite element modelling, published true stress-plastic strain curves should be prioritised. When only engineering stress–strain curves are available, they can be converted to true stress–strain values over the uniform-deformation range before necking occurs. Elastic strain should then be subtracted to obtain true plastic strain. After necking begins, the conventional conversion is no longer sufficient, and constitutive data should be obtained using local strain measurements, inverse identification, or an appropriate post-necking correction [70].

### 6.4. Effects of Defects and Build Orientation

The defect framework developed in Section 5 explains why discontinuities influence tensile properties unevenly. Small, isolated pores may have little effect on yield strength, which is governed mainly by the material’s load-bearing matrix, whereas a large, sharp or connected defect can trigger premature localisation and strongly reduce elongation and fracture strain. Gong et al. [57] found irregular lack-of-fusion defects more damaging than rounded pores, and Montalbano et al. [61] showed that tensile behaviour reflects the combined effects of defect morphology and microstructure rather than total porosity alone. Build orientation modifies this response by changing texture, prior-β grain alignment, layer arrangement, residual stress, surface condition and the orientation of planar defects relative to the loading direction. Consequently, the effect is often clearer in elongation and fracture modes than in yield strength [10,32,33].

### 6.5. Interpretation and Reporting of Tensile Data

Meaningful comparison between articles, therefore, requires tensile data to be grouped by complete material condition. At minimum, reports should distinguish Grade 5 and Grade 23 material used, as-built, stress-relieved, annealed and HIP-treated conditions, machined or as-built surface states, and horizontal, vertical or inclined build orientations. Specimen geometry, strain measurement method and the availability of a complete stress–strain curve should also be stated alongside strength and elongation values. Studies containing full stress–strain curves should be weighted more strongly for constitutive use than those reporting only yield strength, ultimate tensile strength and elongation. The representative results in Table 3 illustrate condition dependence and should be read as condition-specific examples rather than as a combined, universal LPBF Ti6Al4V material model.

## 7. Fatigue Behaviour of LPBF Ti6Al4V

The fatigue performance of LPBF Ti6Al4V can vary widely because crack initiation is highly sensitive to the surface and defect population, while the measured fatigue life also depends on post-processing, orientation, stress ratio, loading mode and specimen size [12,13,50,71].

### 7.1. Comparing Fatigue Datasets and Stress-Life Response

Published LPBF Ti6Al4V fatigue datasets include rotating-bending tests [14], axial and four-point-bending tests [16], and combined axial-torsional tests [72]. These loading modes produce different stress distributions and highly stressed volumes, so their results cannot be ranked without matching the specimen and test conditions. The stress ratio and the stress definition introduce additional distinctions. Fully reversed results at *R* = −1 should not be compared directly with tension-tension results at *R* = 0.1 without an appropriately validated mean-stress correction. Similarly, results expressed in terms of maximum stress should not be ranked directly against those expressed in terms of stress amplitude [71]. Run-outs must also be distinguished from failures. Unless a horizontal asymptote has been demonstrated, a value reported at 10^7^ cycles should be described as fatigue strength at 10^7^ cycles rather than as a true fatigue limit. Continued reductions in fatigue strength have been reported in the very-high-cycle regime [71].

The Basquin relation commonly represents stress-controlled S–N curves:

(4) $$\sigma_{a}=\sigma_{f}^{\prime }\cdot {\left(2\cdot N_{f}\right)}^{b}$$

where *σ_a_* is the stress amplitude, $\sigma_{f}^{\prime }$ is the fatigue strength coefficient, *b* is the fatigue strength exponent, and *N_f_* is the number of cycles to failure [15,73]. Coefficients fitted using maximum stress and stress amplitude are not interchangeable unless the stress and life definitions are converted consistently.

**Figure 5:**
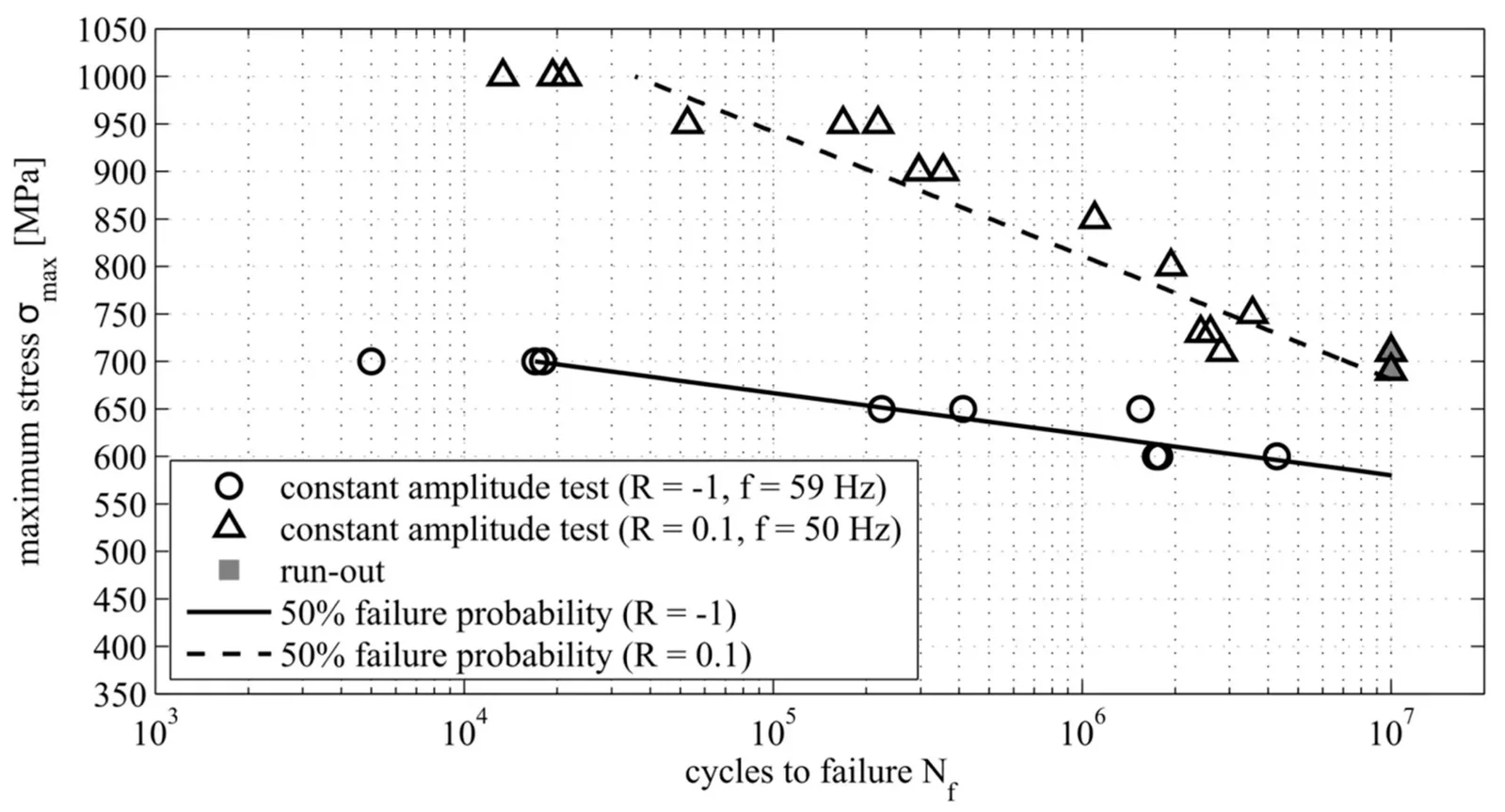
Stress-life response of HIP-treated LPBF Ti6Al4V under tension-tension and fully reversed loading. Reproduced from Wycisk et al. [71] under the Creative Commons Attribution licence.

The magnitude of the surface-condition effect is apparent in multiple studies. Edwards and Ramulu [74] report a substantially reduced fatigue life relative to the conventionally processed reference material under their test conditions. Cutolo et al. [15] directly compared as-produced and machined LPBF specimens under fully reversed axial loading. Wycisk et al. [71] tested machined and polished, stress-relieved and HIP-treated material, with the fully reversed very-high-cycle series extending to 10^9^ cycles. They found that for the HIP-treated specimens, the maximum stress was about 680 MPa at 10^7^ cycles for *R* = 0.1, while the fully reversed stress amplitude decreased from about 575 MPa at 10^7^ cycles to 483 MPa at 10^9^ cycles (Figure 5). These results show why the surface state should be specified in the experimental procedure and why a horizontal fatigue limit should not be assumed.

Accordingly, LPBF Ti6Al4V does not have a single representative fatigue strength. This highlights why data presented in Figure 5 and Table 4 should be interpreted using the complete test definition mentioned above: surface state, post-processing route, stress ratio, loading mode, defect population and life criterion.

### 7.2. Fatigue Initiation: Defects, Orientation, and Post-Processing

**Figure 6:**
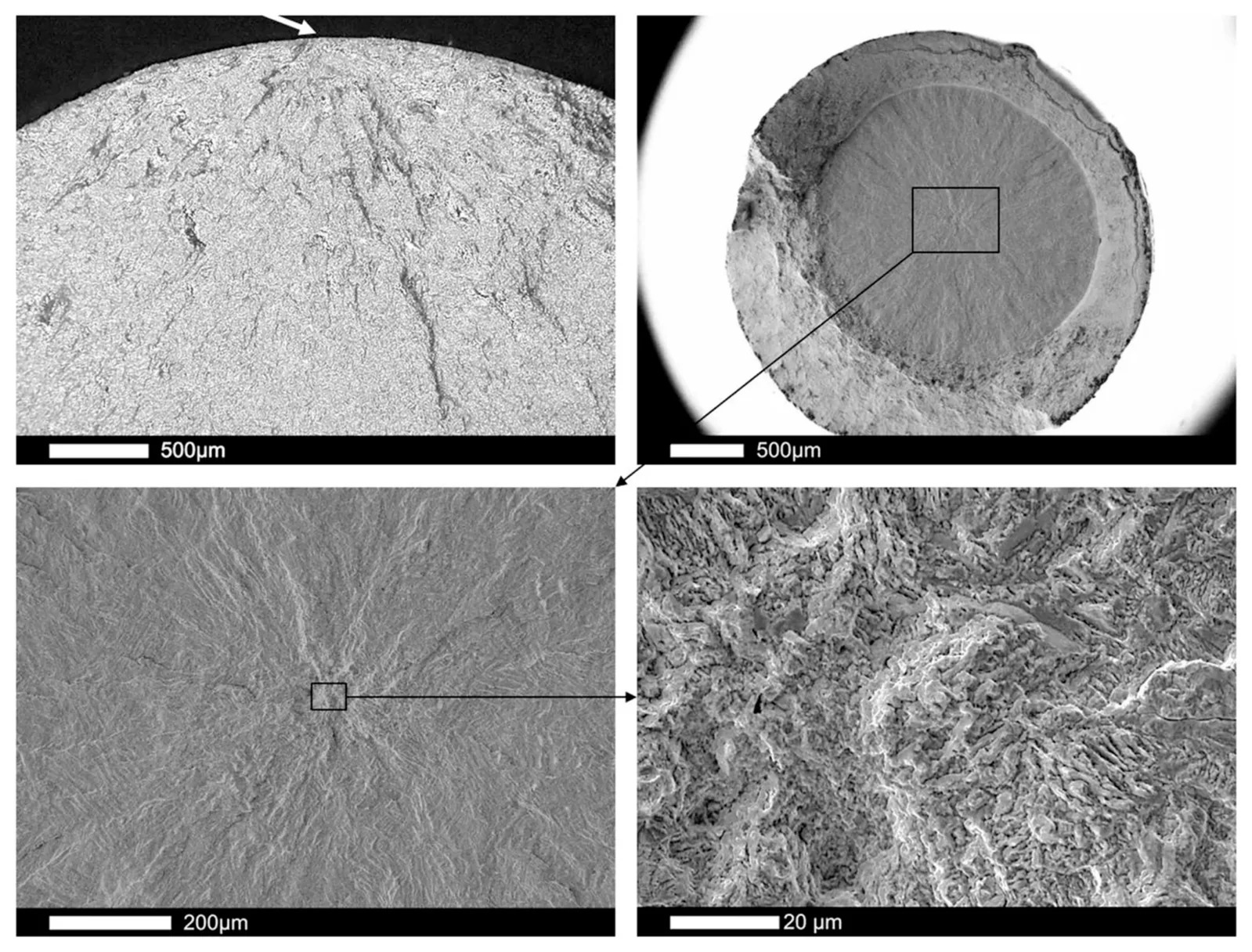
Transition from surface to internal fatigue-crack initiation in HIP-treated LPBF Ti6Al4V. Reproduced from Wycisk et al. [71] under the Creative Commons Attribution licence.

Fatigue life is strongly influenced by the critical initiation site and subsequent small-crack growth, which can involve surface valleys, adhered particles, pores, or near-surface lack-of-fusion defects. The size, shape, and location relationship established in Section 5.2 provides a physical basis for the scatter in fatigue life. Pegues et al. [12] showed that roughness and specimen size both affect fatigue strength. Masuo et al. [50] related fatigue strength to the effective $\sqrt{area}$ parameter, defect location, surface roughness, and HIP treatment. Hu et al. [13] confirmed that near-surface defects are more severe than comparable internal defects. The post-processing techniques described in Section 4 all influence different parts of crack initiation. HIP closes internal enclosed porosity but does not remove the rough surface or reliably close surface defects. To address these surface-controlled initiation sites, machining, polishing, or etching should be used depending on component geometry. In Wycisk et al. [71] for machined and polished HIP specimens, initiation shifted from the surface to the interior at longer lives, as shown in Figure 6.

Build orientation not only alters the alignment of layers and prior-beta grains, but it also changes the surface quality of inclined faces. Because of this, the observed fatigue response depends on surface condition, defects, microstructure, residual stress, and build direction. Sun et al. [35] reported a significant orientation effect on S−N behaviour, but a smaller effect on long-crack propagation. More recent studies provide further insight into the underlying mechanisms. Zhang et al. [77] reported the highest fatigue life for the 75° orientation. Lower build angles were more strongly affected by defects on the downward-facing surface, while pore clustering contributed to the scatter observed at higher angles. Dong et al. [78] showed that the effects of loading orientation depended on the post-processing condition. In the stress-relieved and sub-transus-treated states, fatigue cracks predominantly initiated at manufacturing defects, whereas after HIP, the reduction in the defect population shifted crack initiation toward microstructural facets. These findings indicate that orientation alone is not sufficient to explain the reported fatigue rankings and should be considered alongside surface condition, defect population, and post-processing state.

### 7.3. Very-High-Cycle Fatigue Behaviour

The available evidence does not support a universal horizontal S−N asymptote for LPBF Ti6Al4V. Wycisk et al. [71] observed continued fatigue-strength reduction to 10^9^ cycles and a transition from surface to internal initiation in machined, polished and HIP-treated specimens. The transition indicates that, once surface-controlled initiation is suppressed, fatigue life can become governed by internal defects. Du et al. [79] reported defect-controlled internal VHCF initiation and stress-ratio-dependent rough-area morphology, while Brot et al. [80] showed that either a large pore or a microstructural feature could govern VHCF initiation depending on material condition.

Fractographic studies further show that the morphology surrounding these internal origins depends on the loading and material condition. Sun et al. [81] reported fish-eye-like regions and fine granular areas under ultrasonic loading, whereas the rotating-bending specimens initiated at the surface. Fu et al. [82] reported rough-area and nanograin formation at several stress ratios but not at *R* = 0.5, and Sun et al. [83] later observed fine granular areas around internal lack-of-fusion origins in a deliberately defect-containing SLM condition. These studies show that internal VHCF initiation is not associated with a single universal fracture morphology. Terms such as fish-eye, rough area, fine granular area and facet should instead be used only where the corresponding features were actually observed.

The same hierarchy between defects and microstructure is also evident at shorter fatigue lives. Dong et al. [78] found that HIP shifted crack initiation from process defects toward microstructural facets, while Bonneric et al. [63] distinguished the effects of defect size, type and surface proximity. These HCF studies support the broader interpretation that the dominant crack-initiation mechanism changes with defect population and material condition, but should not be treated as direct evidence of 10^8^–10^9^-cycle behaviour.

### 7.4. Low-Cycle Fatigue and Cyclic Plasticity

When local strain exceeds the elastic regime, as may occur near notches, thin sections, defects or geometric stress concentrations, a strain-life description is more relevant than a stress-life curve. The total strain amplitude may be represented using the Basquin–Coffin–Manson relation [84,85]:

(5) $$\varepsilon_{a}=\frac{\sigma_{f}^{\prime }}{E}{\left({2N}_{f}\right)}^{b}+\varepsilon_{f}^{\prime }{\left({2N}_{f}\right)}^{c}$$

where $\varepsilon_{a}$ is the total strain amplitude, $\varepsilon_{f}^{\prime }$ is the fatigue ductility coefficient, and *c* is the fatigue ductility exponent. The corresponding stabilised cyclic stress–strain response is described by the cyclic Ramberg–Osgood relation:

(6) $$\varepsilon_{a}=\frac{\sigma_{a}}{E}+{\left(\frac{\sigma_{a}}{K^{\prime }}\right)}^{\frac{1}{n^{\prime }}}$$

where *K*′ is the cyclic strength coefficient and *n*′ is the cyclic strain hardening exponent. These relationships must be fitted to a defined material condition and strain range. Zhang et al. [84] tested machined and polished, thermally untreated and annealed, LPBF Ti6Al4V samples at strain amplitudes of 1.2%, 1.4%, 1.6%, and 2.0% and observed cyclic softening in both the as-built and annealed states. The comparison with wrought material depended on strain amplitude and material condition. Xi et al. [85] reported additional strain-life parameters for heat-treated and HIP-ed conditions, while Goyal et al. [86] quantified cyclic softening in annealed LPBF Ti6Al4V ELI. These parameter sets should remain associated with their material condition and fitting conventions. Extrapolation beyond the tested range requires validation. Table 5 collects representative parameter sets.

### 7.5. Mean-Stress and Multiaxial Loading

The mean stress affects both the maximum stress and the degree of crack opening, so tensile mean stress regimes usually reduce fatigue life. In LPBF materials, however, the apparent sensitivity also depends on whether the critical defect is internal or at the surface [71,73]. Any mean-stress correction applied to LPBF Ti6Al4V should be calibrated and validated using data obtained under comparable material, surface, defect, and loading conditions and at more than one stress ratio [71,73]. The same validation should apply under multiaxial loading. Components may experience combined axial, bending, shear and torsional stresses. Fatemi et al. [72] demonstrated that surface finishing remains influential in both uniaxial and multiaxial conditions. They also found different governing multiaxial damage descriptions for AM and wrought material. More recent multiaxial experiments on unmachined LPBF Ti6Al4V have similarly shown that build orientation and surface micro-notches affect fatigue life, and that cracking can be governed by the maximum tensile-stress plane [87]. Although the available multiaxial database has expanded, it remains too limited in material condition, surface state and loading-path coverage to support a general parameter recommendation for LPBF Ti6Al4V.

### 7.6. Small- and Short-Crack Behaviour

Manufacturing defects can act as effective initial cracks, but the earliest propagation stage does not necessarily follow long-crack threshold or Paris-law behaviour. Waddell et al. [88] used in situ synchrotron X-ray tomography and diffraction to observe small-crack growth in SLM Ti6Al4V. In their trial build, nearby pores blunted or locally retarded the three-dimensional crack front, and the measured small-crack rates were below the corresponding long-crack expectation. The result demonstrates that defect interaction is condition-dependent rather than universally accelerating every small crack.

Wu et al. [89] used an artificial semi-elliptical defect in LPBF Ti6Al4V and found that small cracks could propagate below the conventional long-crack threshold and faster than long cracks at the same ∆*K*. Shamir et al. [90] used WAAM Ti6Al4V, rather than LPBF material, to validate a modified Hartman−Schijve representation with a small-crack threshold. That result supports process-different evidence and is not pooled with the LPBF data. Overall, the available evidence suggests that long-crack and Paris-law parameters should not be applied directly from the onset of defect-controlled crack growth.

The remaining gap is not a complete absence of short-crack research, but rather the lack of condition-specific transitions covering natural lack-of-fusion, gas-pore, near-surface, and surface-breaking defects across alloy grades, surface states, post-processing conditions, and stress ratios.

### 7.7. Long-Crack Fatigue-Growth Behaviour

Conventional da/dN-∆K data primarily describe physically long cracks after a defect-initiated crack has reached a scale at which continuum fracture-mechanics assumptions are appropriate. They should therefore be distinguished from both initiation-controlled S−N data and the small-crack behaviour discussed in Section 7.6. Leuders et al. [49] reported lower applied long-crack thresholds in as-built specimens than in heat-treated or HIP-treated conditions. Representative long-crack and near-threshold values are listed in Table 6.

These values describe long-crack or near-threshold behaviour and should not be compared across studies unless the stress ratio, crack-closure treatment, residual stress, orientation, microstructure, test method, and threshold definition are taken into account. Leuders et al. [49] reported applied long-crack thresholds, whereas Becker et al. [40] reported an intrinsic, crack-closure-corrected threshold. The latter is not directly equivalent to ASTM load-shedding values. Sun et al. [35] reported a comparatively small orientation effect on long-crack growth under the conditions investigated.

Paul et al. [93] showed that stress ratio and notch orientation affected threshold and crack-growth behaviour, whereas process-induced volumetric defects did not significantly alter stable crack growth under the tested conditions. This reinforces the need to separate initiation-controlled S−N data from crack-growth data and to report the crack-growth orientation and load ratio.

## 8. Discussion

### 8.1. Sources of Variability Between Published Results

The reviewed studies show that LPBF Ti6Al4V does not represent a single material condition. Table 7 provides selected quantitative comparisons of alloy grade, surface condition, powder reuse and manufacturing defect populations under defined experimental conditions. These comparisons demonstrate how differences in material state and processing can affect fatigue performance, while loading mode, stress ratio and life criterion determine whether results from different studies can be compared directly.

Powder reuse adds an upstream source of variation that is easy to overlook. The reviewed LPBF evidence indicates that reuse can alter oxygen content, particle morphology, flowability and packing before any change in the consolidated material is measured [27,28]. These changes can then alter melt stability and the defect population. Consequently, reuse history and feedstock management should be treated as part of the material condition, but a fatigue penalty should not be assumed without condition-specific fatigue data.

### 8.2. Interaction Between Microstructure, Defects and Surface Condition

The relative importance of microstructure and defects changes with material quality. In rough as-built specimens, surface valleys and surface-connected defects can dominate crack initiation even when the matrix has high tensile strength [12,50]. After surface-controlled initiation is suppressed, internal defects can become more important [71]. Consequently, improvements in static strength do not necessarily produce comparable improvements in fatigue performance.

Residual stress participates in the same hierarchy. A tensile near-surface field can increase local crack driving force, but its apparent importance diminishes after thermal stress relief or when a dominant defect produces a larger local concentration [41]. Limited mechanical loading can redistribute residual stress [42], but cycle-resolved relaxation during representative LPBF Ti6Al4V fatigue has not been established. In VHCF, suppressing rough-surface initiation can expose internal defect- or microstructure-controlled mechanisms [71,80]. HCF studies further show that the dominant crack-initiation mechanism can change with post-processing and defect population [63,78].

Initiation and propagation must also be connected. Roughness or a surface-breaking defect often controls the initial crack, but internal defects and microstructure become more visible after surface initiation sites are reduced. During early growth, a small physical crack can interact with neighbouring pores and the local microstructure [88,89]. Long-crack thresholds and Paris-law constants become appropriate only after the crack has transitioned to a scale for which continuum fracture mechanics is valid.

### 8.3. Benefits and Trade-Offs of Post-Processing

The post-processing routes reviewed in Section 4 act on different damage mechanisms and should not be treated as interchangeable. Stress relief and annealing primarily modify residual stress and microstructure. HIP additionally targets enclosed internal porosity, but can change the α-lath morphology, and surface finishing removes or blunts external initiation sites without eliminating internal defects. Consequently, the largest fatigue benefit is expected when the dominant defect population is identified first, and the treatment route is matched to that mechanism [45,46].

This mechanism-based view also explains why HIP can be highly beneficial in one study but less effective in another. For example, Masuo et al. [50] reported an approximately 40–65 MPa increase in fatigue strength following HIP when the rough surface was retained, compared with approximately 240 MPa for polished specimens under their investigated rotating-bending conditions (*R* = −1, approximately 10^7^ cycles). This illustrates that the benefit of reducing internal porosity can be limited when surface-controlled crack initiation remains dominant. If a rough surface, a large surface-connected cavity, or an inaccessible downward-facing region remains, surface initiation can continue to govern despite internal densification [55,56]. Conversely, once HIP reduces the influence of manufacturing defects, microstructural features can become increasingly important in controlling fatigue performance. Dong et al. [78] observed a shift from defect-controlled initiation to microstructural-facet initiation after HIP. Post-processing should therefore be evaluated as a sequence targeting the controlling initiation site, rather than as a generic material upgrade.

### 8.4. Comparability of Fatigue Data

The spread of published fatigue values is due in part to physical factors and in part to methodological factors. Axial and bending tests sample different highly stressed volumes, positive mean stress changes crack opening, and maximum stress and stress-amplitude conventions cannot be combined without conversion. In addition, a run-out at a prescribed cycle count is not evidence of a true fatigue limit. The practical implication is that pooled fatigue curves are defensible only when material condition, surface state, loading mode, stress ratio, stress convention and life criterion are sufficiently consistent.

Orientation-dependent fatigue results illustrate this comparability problem. A change in build or loading orientation can change several material and geometric variables, making orientation effects difficult to isolate across studies. Dong et al. [78] showed that the influence of loading orientation changed with post-processing. Zhang et al. [77] reported approximately 4.5 × 10^4^ cycles for the 0° orientation and 1.3 × 10^5^ cycles for the 75° orientation at σ_max_ = 400 MPa and *R* = 0.1, corresponding to an approximately threefold difference in fatigue life. Simonelli et al. [10] reported tensile elongations of 12.4% and 7.0% for the stress-relieved edge and flat orientations, respectively, while their UTS values differed by only 8 MPa (Table 3). These results illustrate that orientation affects tensile and fatigue properties differently, and that the observed differences also depend on surface condition, defect population, and post-processing.

### 8.5. Remaining Knowledge Gaps

The main gaps concern direct cycle-resolved residual-stress measurements during conventional fatigue, condition-specific strain-life parameters for realistic as-built surfaces, multiaxial loading with representative surface states, and validated transitions from manufacturing-defect-controlled small-crack growth to long-crack propagation. Additional VHCF experiments should resolve surface-to-internal initiation across controlled surface and defect conditions. Recently published specimen-level fatigue datasets also provide opportunities for more systematic statistical comparisons of defect populations and fatigue scatter, particularly when material condition, defect characteristics and loading conditions are recorded consistently. For example, Ley et al. [16] provide 175 axial/four-point-bending test records for stress-relieved, low-stress-ground Ti6Al4V at *R* = 0.1 and 20 Hz, including initiation-site characterisation for 136 failed records. Powder reuse studies should link measured chemistry and powder-bed changes directly to fatigue response, and dense-coupon parameters should be validated before transfer to geometrically complex LPBF structures.

## 9. Conclusions

LPBF Ti6Al4V does not have a universal set of mechanical or fatigue properties. Alloy grade and interstitial chemistry influence the matrix response, while thermal history, microstructure, residual stress, defect morphology and location, surface condition and post-processing determine where deformation and fatigue damage develop. Stress ratio, loading mode, stressed volume, stress definition and run-out criterion further control the reported fatigue results. HIP is most effective when enclosed internal pores are limiting and cannot replace surface finishing when rough or surface-connected features control initiation.

The review’s contribution is a condition-specific quantitative comparison of tensile properties, stress-life data, cyclic stress–strain and strain-life parameters, and long-crack growth data, together with the metadata required for engineering transfer. The remaining priorities are cycle-resolved residual-stress relaxation, fatigue parameters for realistic as-built surfaces and multiaxial loading, short-to-long-crack transition data for natural manufacturing defects, systematic VHCF surface-to-internal transition studies, and validation of coupon-derived parameters for complex LPBF components.

## Figures and Tables

**Table 1:** Chemical composition limits (wt.%) of elements in Ti6Al4V based on ASTM F2924 [22] for Grade 5 and F3001 [21] for Grade 23 ELI Ti6Al4V.

Element	Grade 5 (Ti6Al4V)	Grade 23 (Ti6Al4V ELI)
Aluminium (Al)	5.5–6.75%	5.5–6.50%
Vanadium (V)	3.5–4.5%	3.5–4.5%
Oxygen (O)	≤0.20%	≤0.13%
Nitrogen (N)	≤0.05%	≤0.05%
Iron (Fe)	≤0.30%	≤0.25%
Carbon (C)	≤0.08%	≤0.08%
Hydrogen (H)	≤0.015%	≤0.012%
Titanium (Ti)	Balance	Balance

**Table 2:** Direct comparison of the principal effects of common post-processing routes applied to LPBF Ti6Al4V.

Treatment	Porosity	Microstructure	Residual Stress	Surface Condition	Fatigue Effect
Stress relief	Little direct effect	Limited tempering/decomposition, depending on cycle	Reduced	Generally unchanged	Benefit depends on remaining defects and surface condition
Annealing/heat treatment	Limited pore closure	Strong α′ decomposition and possible α-lath coarsening	Reduced	Generally unchanged	Can improve ductility. Fatigue benefit depends on defect and surface control
HIP	Closes suitable enclosed internal pores	α′ transformation, possible α-lath coarsening	Reduced	Rough and surface-connected defects remain	Large benefit when internal porosity is critical, and surface is subsequently controlled
Surface finishing	Internal pores largely unchanged	Usually limited bulk effect	Process-dependent, peening may introduce compressive stress	Improved	Strong benefit when surface-controlled crack initiation dominates

**Table 3:** Representative quasi-static tensile properties of LPBF Ti6Al4V under different material, build-orientation, and post-processing conditions. NR: not reported.

Reference	Material and Condition	Build Orientation	E [GPa]	0.2% Yield Strength [MPa]	UTS [MPa]	Elongation/Fracture Strain [%]
[14]	Grade 23 ELI, as-built	Vertical	NR	1167.1 ± 4.5	1295.8 ± 3.5	4.6 ± 0.7
[14]	Grade 5, as-built	Vertical	NR	1225.7 ± 7.2	1360.9 ± 5.5	3.1 ± 1.0
[14]	Grade 23 ELI, HIP: 920 °C, 102 MPa, 2 h	Vertical	NR	887.6 ± 8.1	996.0 ± 1.1	13.8 ± 0.6
[14]	Grade 5, HIP: 920 °C, 102 MPa, 2 h	Vertical	NR	937.1 ± 9.9	1032.5 ± 2.4	14.4 ± 0.5
[43]	Untreated SLM; Grade 23; wire-EDM-cut specimen	Loading perpendicular to the build direction	109.2 ± 3.1	1110 ± 9	1267 ± 5	7.28 ± 1.12
[43]	Heat treated at 850 °C for 2 h; furnace cooled; Grade 23; wire-EDM-cut specimens	Loading perpendicular to the build direction	114.7 ± 3.6	955 ± 6	1004 ± 6	12.84 ± 1.36
[43]	Heat treated at 1020 °C for 2 h; furnace cooled; Grade 23; wire-EDM-cut specimens	Loading perpendicular to the build direction	114.7 ± 0.9	760 ± 19	840 ± 27	14.06 ± 2.53
[10]	As-built; mechanically polished	Edge, xz	115 ± 6	978 ± 5	1143 ± 6	11.8 ± 0.5
[10]	As-built; mechanically polished	Vertical, zx	119 ± 7	967 ± 10	1117 ± 3	8.9 ± 0.4
[10]	As-built; mechanically polished	Flat, xy	113 ± 5	1075 ± 25	1199 ± 49	7.6 ± 0.5
[10]	Stress relieved at 730 °C for 2 h; mechanically polished	Edge, xz	113 ± 9	958 ± 6	1057 ± 8	12.4 ± 0.7
[10]	Stress relieved at 730 °C for 2 h; mechanically polished	Vertical, zx	117 ± 6	937 ± 9	1052 ± 11	9.6 ± 0.9
[10]	Stress relieved at 730 °C for 2 h; mechanically polished	Flat, xy	112 ± 6	974 ± 7	1065 ± 21	7.0 ± 0.5

**Table 4:** Representative source-verified HCF and VHCF results for LPBF Ti6Al4V.

Reference	Grade/Oxygen	Build Orientation	Surface and Post-Processing	Loading Mode, R, Frequency	Specimen Geometry	Life Criterion/Run-Out Definition	Reported Fatigue Result and Stress Definition
[71]	NR/NR	90° (upright)	Machined and polished; HIP 920 °C, 100 MPa, 2 h	Axial; R = 0.1; 50 Hz	Cylindrical	Fatigue strength at 10^7^ cycles; termination/run-out rule NR	$\sigma_{max}\approx 680\;MPa$
[71]	NR/NR	90° (upright)	Machined and polished; HIP 920 °C, 100 MPa, 2 h	Axial; R = −1; 59 Hz	Cylindrical	Fatigue strength at 10^7^ cycles; termination/run-out rule NR	$\sigma_{max}\approx 575\;MPa$
[71]	NR/NR	90° (upright)	Machined and polished; HIP 920 °C, 100 MPa, 2 h	Axial; R = −1; 20 kHz (pulse-pause)	Ultrasonic, resonance specimen	Fatigue strength at 10^9^ cycles; termination/run-out rule NR	$\sigma_{max}\approx 483\;MPa$
[49]	O = 0.17 wt.% after SLM	Z-direction	Machined from cylindrical SLM rods; untreated material condition	Uniaxial; R = −1; 40 Hz	Cylindrical, ASTM E466-07 [75]	Cycles to failure at fixed stress amplitude	$\sigma_{a}=600\;MPa$ $N_{f}=2.7\times 10^{4}$
[49]	O = 0.17 wt.% after SLM	Z-direction	Machined; 800 °C, 2 h, Ar, furnace cooled	Uniaxial; R = −1; 40 Hz	Cylindrical, ASTM E466-07	Cycles to failure at fixed stress amplitude	$\sigma_{a}=600\;MPa$ $N_{f}=9.3\times 10^{4}$
[49]	O = 0.17 wt.% after SLM	Z-direction	Machined; 1050 °C, 2 h, vacuum, furnace cooled	Uniaxial; R = −1; 40 Hz	Cylindrical, ASTM E466-07	Cycles to failure at fixed stress amplitude	$\sigma_{a}=600\;MPa$ $N_{f}=2.9\times 10^{5}$
[49]	O = 0.17 wt.% after SLM	Z-direction	Machined; 920 °C, 100 MPa 2 h, Ar	Uniaxial; R = −1; 40 Hz	Cylindrical, ASTM E466-07	No failures at 600 MPa by 2 × 10^6^	$\sigma_{a}=600\;MPa$, all run-outs to 2 × 10^6^
[46]	NR/NR	NR	HIP 930 °C, 100 MPa, 4 h + chemical etching	Four-point bending; R = 0.1; ≈70 Hz	10 mm × 10 mm × 80 mm	Run-out defined as 10^7^ cycles	Highest run-out strength $\sigma_{max}\approx 450\;MPa$ at 10^7^ cycles
[76]	Powder O = 0.149 wt.%	Rectangular samples printed in diamond orientation	As-built surface; stress relieved 600 °C, 120 min, vacuum; EOS nominal-improved condition	Four-point bending; R = 0.1; 10 Hz	5 mm × 5 mm × 70 mm	Approx. 10^7^ cycles to failure; not reported as a formal run-out limit	$\sigma_{a}\approx 300\;MPa\;at\;∼\;10^{7}cycles$

NR denotes information that is not reported in the cited text. The values are finite-life fatigue strengths at the stated cycle count, not automatically fatigue limits. Rows with different stress ratios, stress definitions, loading modes, surface states, specimen geometries or frequencies should not be ranked against each other directly.

**Table 5:** Representative cyclic Ramberg–Osgood and Basquin–Coffin–Manson parameters for LPBF Ti6Al4V under different heat-treatment conditions.

Material Condition	Monotonic E [GPa]	K′ [MPa]	n′	σ′*_f_* [MPa]	b	ε′*_f_*	c	Reference
As-built SLM; machined and polished	106	1535.15	0.07833	2452.03	−0.09165	7.7848	−1.14246	[84]
Annealed SLM, 800 °C for 2 h; machined and polished	109	1222.59	0.05986	2017.46	−0.08428	1.0541	−0.85695	[84]
Heat-treated SLM: 500 °C for 0.5 h, followed by 800 °C for 4 h	116.2	1311.6	0.0695	1302	−0.0509	0.236	−0.582	[85]
HIP-treated SLM: 920 °C, 120 MPa for 2 h	118.6	1234.2	0.0674	1076	−0.0351	0.177	−0.453	[85]
LPBF Ti6Al4V ELI; annealed at 900 °C for 2 h; vertically built, machined and polished	117.6	1499.9	0.0887	1465.42	−0.0763	0.1832	−0.5363	[86]
Wrought reference	110	-	-	2030	−0.104	0.841	−0.69	Suresh, as reproduced in [84]

**Table 6:** Reported fatigue-crack-growth values for LPBF Ti6Al4V under different material conditions and crack-growth orientations.

Reference	Material Condition	Crack-Growth Direction/Orientation	Loading Condition	ΔK*_th_* [MPa√m]
[49]	As-built SLM	Crack growth perpendicular to the build direction	Compact-tension specimen; (*R* = 0.1); ASTM E647 [91] load shedding	1.4
[49]	As-built SLM	Crack growth parallel to the build direction	Compact-tension specimen; (*R* = 0.1); ASTM E647 load shedding	1.7
[49]	Heat-treated/HIP-treated conditions combined	Crack growth perpendicular to the build direction	*R* = 0.1	3.9 ± 0.4
[49]	Heat-treated/HIP-treated conditions combined	Crack growth parallel to the build direction	*R* = 0.1	4.6 ± 0.9
[40]	As-fabricated	Three compact-tension specimen orientations	Multiple load ratios; residual-stress and crack-closure analysis	Intrinsic ΔK*_th_* ≈ 1.6 ± 0.2
[92]	Flat-top-beam LPBF; as-built	Horizontal-built CT blanks; see source Figure 2	CT; R = 0.1; 15 Hz; load shedding; da/dN = 10^−7^ mm/cycle	≈3.7
[92]	Flat-top-beam LPBF; 850 °C/2 h; furnace cooled	Horizontal-built CT blanks; see source Figure 2	CT; *R* = 0.1; 15 Hz; load shedding; da/dN = 10^−7^ mm/cycle	5.5
[92]	Flat-top-beam LPBF; 850 °C/2 h; water cooled; 540 °C/4 h; air cooled	Horizontal-built CT blanks; see source Figure 2	CT; *R* = 0.1; 15 Hz; load shedding; da/dN = 10^−7^ mm/cycle	6.3

**Table 7:** Selected within-study quantitative comparisons for LPBF Ti6Al4V.

Comparison	Common Conditions	Reported Result	Ref.
Grade/chemistry	Smooth, machined and HIP-treated, rotating bending, *R* = −1; 10^7^ cycles	Grade 23 ≈ 595 MPa; Grade 5 ≈ 645 MPa (σ_a_); ≈50 MPa difference. Bulk O: 0.110 and 0.164 wt.%, respectively	[14]
Surface/orientation	Grade 23; heat treated at 850 °C/2 h; axial loading, *R* = −1; termination at 2 × 10^6^	As-produced → machined run-out stress levels (σ_a_): 0°: 166.5 MPa → 575 MPa; 90°: 225.5 MPa → 530 MPa	[15]
HIP × surface	Rotating bending, *R* = −1; approximately 10^7^ cycles	Approximate HIP-associated fatigue-strength increase: 40–65 MPa with rough surfaces retained; ≈240 MPa for polished specimens	[50]
Processing/defect population	Different processing conditions; nominal porosity ≈ 5 vol.%, *R *= 0.1, 10^7^ cycles	Approximate reported fatigue stress levels: σ_max_ ≈ 300 MPa for rounded-pore-rich and σ_max_ ≈ 100 MPa for LOF-rich conditions. Processing parameters also differed	[57]
Powder reuse	Machined Grade 5; 704 °C/1h; σ_a_ = 600 MPa, R = −1, 4 Hz; four failures per group	Mean N_f_ (fresh/3/7 reuses): north 4.0/9.3/2.7 × 10^5^; south 3.1/1.2/0.82 × 10^5^ cycles	[27]

## Data Availability

No new data were created or analysed in this study. Data sharing is not applicable to this article.

## Abbreviations/Nomenclature

Symbol/Abbreviation	Definition
AM	Additive manufacturing
ELI	Extra-low-interstitial
HCF	High-cycle fatigue
HIP	Hot isostatic pressing
LCF	Low-cycle fatigue
*N*_f_	Number of cycles to failure
*R*	$\mathrm{Stress}\;\mathrm{ratio},\;\sigma_{min}$ $/\sigma_{max}$
SLM	Selective laser melting
UTS	Ultimate tensile strength
VHCF	Very-high-cycle fatigue
YS	Yield strength
$\sqrt{area}$	Square root of the defect area
α	Alpha phase
α′	Martensitic alpha-prime phase
β	Beta phase
*E*	Young’s modulus
$\sigma_{a}$	Stress amplitude
$\sigma_{min}$	Minimum stress
$\sigma_{max}$	Maximum stress
$\sigma_{res}$	Residual stress
∆σ	Stress range
∆ε	Total strain range
∆*K*	Stress-intensity-factor range
∆*Kth*	Fatigue-crack-growth threshold
d*a*/d*N*	Fatigue-crack-growth rate
*K*′	Cyclic strength coefficient
*n*′	Cyclic strain-hardening exponent
$\sigma_{f}^{\prime }$	Fatigue strength coefficient
$\varepsilon_{f}^{\prime }$	Fatigue ductility coefficient
*b*	Fatigue strength exponent
*c*	Fatigue ductility exponent
CT	Compact tension
